# Supplementary material for: Identification of photoperiod-induced specific miRNAs in the adrenal glands of Sunite sheep (Ovis aries)
Source: Front Vet Sci. 2022 Jul 22;9:888207. doi: 10.3389/fvets.2022.888207 (PMC9354845; doi:10.3389/fvets.2022.888207)
Supplement: Supplementary file 1 [file Data_Sheet_1.ZIP › Supplementary file/miRDeep2 file.zip!/miRDeep2 file/Know_Structure/LP42A3/oar-mir-411b.pdf]

miRBase precursor : oar-mir-411b
Total read count : 5388
oar-miR-411b-5p read count : 5301
oar-miR-411b-3p read count : 87
remaining reads : 0

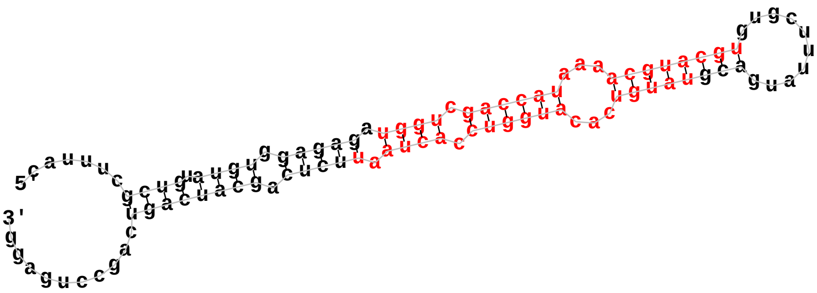

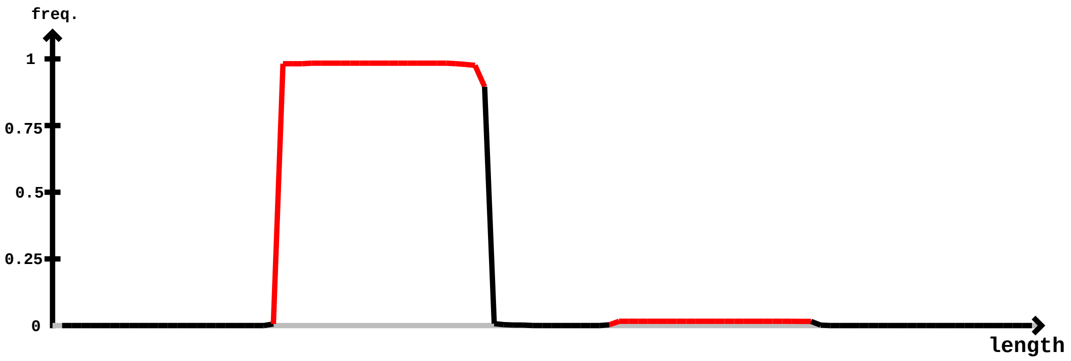


```
                                                                              oar-miR-411b-3p
                         oar-miR-411b-5p
5'- cauuucgcuguauguggagagauggucgaccauaaaacguacgugugcuuuaugacguaugucacaugguccacuaauucucagcaucagucagccugagg  -3'   exp
    ......((((.((((.(((((.((((.((((((...((((((((..........))))))))...)))))).)))).))))).)))))))).........    reads   mm   sample
    .....................auggucgaccauaaaacgu..............................................................    1       0    seq
    .....................auggucgaccauaaaacguacg...........................................................    29      0    seq
    .....................auggucgaccauaaaacguacgu.........................................................    2       0    seq
    ......................uggucgaccauaaaacgu..............................................................    9       0    seq
    ......................uggucgaccauaaaacgua............................................................    15      0    seq
    ......................uggucgaccauaaaacguac...........................................................    19      0    seq
    ......................uggucgaccauaaaacguacg..........................................................    403     0    seq
    ......................uggucgaccauaaaacguacgu.........................................................    4784    0    seq
    ......................uggucgaccauaaaacguacgug........................................................    20      0    seq
    ......................uggucgaccauaaaacguacgugu.......................................................    8       0    seq
    ........................ucgaccauaaaacguacgugug.......................................................    1       0    seq
    ........................ucgaccauaaaacguacgugugc......................................................    9       0    seq
    ........................ucgaccauaaaacguacgugugcu.....................................................    1       0    seq
    ........................................................guaugucacaugguccacu..........................    1       0    seq
    ........................................................guaugucacaugguccacua.........................    1       0    seq
    ........................................................guaugucacaugguccacuaau.......................    14      0    seq
    .........................................................uaugucacaugguccacua.........................    1       0    seq
    .........................................................uaugucacaugguccacuaau.......................    61      0    seq
    .........................................................uaugucacaugguccacuaauu......................    9       0    seq
```
